# One Velocity Loss Threshold Does Not Fit All: Consideration of Sex, Training Status, History, and Personality Traits When Monitoring and Controlling Fatigue During Resistance Training

**DOI:** 10.1186/s40798-023-00626-z

**Published:** 2023-09-05

**Authors:** Ivan Jukic, Katarina Prnjak, Michael R. McGuigan, Eric R. Helms

**Affiliations:** 1 Sport Performance Research Institute New Zealand (SPRINZ), Auckland University of Technology, Auckland, New Zealand; 2 School of Engineering, Computer and Mathematical Sciences, Auckland University of Technology, Auckland, New Zealand; 3 School of Medicine, Western Sydney University, Sydney, Australia

**Keywords:** Velocity-based training, Strength training, Exercise monitoring, Exercise prescription

## Abstract

**Purpose:**

This study aimed to quantify the potential variability in the volume of work completed after reaching different velocity loss (VL) thresholds and determine the effects of sex, training status and history, as well as psychological traits on the reliability and magnitude of the amount of work completed after reaching different VL thresholds using different loads in the back-squat exercise.

**Methods:**

Forty-six resistance-trained people (15 females and 31 males; 18 to 40 years of age) with a wide range of strength levels, training experience, and different training practices were recruited and performed a one-repetition maximum (1RM) test, and two repetitions to failure (RTF) tests 72 h apart. RTF tests were performed with 70, 80, and 90% of 1RM with 10 min of rest between sets. The Bland–Altman analysis for multiple observations per participant and equivalence tests were used to quantify the variability in the volume of work completed after reaching different VL thresholds, whereas linear and generalised mixed-effects models were used to examine the effects of different moderators on the stability and magnitude of the amount of work completed after reaching different VL thresholds.

**Results:**

The findings of the present study question the utility of using VL thresholds to prescribe resistance training (RT) volume as the agreement in the amount of work completed across two consecutive testing sessions was not acceptable. Regardless of the load used, females completed more repetitions than males across VL thresholds, while males performed repetitions at higher velocities. In addition, individuals with higher levels of emotional stability also tended to perform more repetitions across VL thresholds. Finally, sex, choice of load, strength levels and training practices, as well as emotional stability affected the linearity of the repetition–velocity relationship and when sets terminated.

**Conclusion:**

Using the same VL thresholds for all individuals, while assuming generalisability of the stimuli applied, would likely lead to variable acute physiological responses to RT and divergent neuromuscular adaptations over long term. Therefore, VL monitoring practices could be improved by considering sex, training status, history, and psychological traits of individuals due to their effects on the variability in responses to different VL thresholds.

**Supplementary Information:**

The online version contains supplementary material available at 10.1186/s40798-023-00626-z.

## Key Points

- This study comprehensively examined the stability and magnitude of work completed across velocity loss thresholds during resistance training with free-weights while also exploring the effects of several factors potentially influencing the use of velocity loss thresholds in practice.
- Velocity loss thresholds cannot be used to reliably control the amount of work completed during resistance training, but rather as a general variable quantifying the amount of fatigue experienced by an individual.
- Velocity loss thresholds should be highly individualised when used in practice since load, sex, training status, history, and personality traits can all influence, at least to some extent, the variability in responses to different velocity loss thresholds.

**Supplementary Information:**

The online version contains supplementary material available at 10.1186/s40798-023-00626-z.

## Background

Resistance training (RT) is widely recognised as an effective and efficient mode of training for stimulating a vast array of neuromuscular adaptations. There are many variables that play a part in designing an efficacious RT program such as training load, volume and frequency, rest periods, exercise type, movement velocity, and set structure configuration [1]. Of these, training volume has received the most attention from the scientific community, and it is often seen as one of the key drivers of RT-induced adaptations such as muscle strength and hypertrophy [2, 3]. Traditionally, RT set volume is prescribed according to a theoretical maximum number of repetitions that can be performed against a given percentage of one-repetition maximum (%1RM) until reaching muscular failure (e.g. 10 repetitions with 75% of 1RM). Based on this, a specific number of repetitions are often prescribed for a group of individuals using the same relative load for all exercises. However, this method of prescribing RT volume might not provide the planned training stimulus for all individuals since the number of repetitions that can be completed against a given %1RM is both individual- and exercise-specific [4, 5]. For instance, if all individuals perform the same number of repetitions per set against the same relative load, it is possible that they will experience different degrees of fatigue upon completing a set, as the number of repetitions left in reserve could be considerably different between individuals. To combat these issues, instead of performing a fixed, predetermined number of repetitions, researchers have suggested terminating each training set as soon as a predetermined percentage of velocity loss (VL) is reached [5, 6].

Several studies have shown that monitoring VL is an objective, practical, and non-invasive indicator of the acute metabolic stress, hormonal response, and mechanical fatigue induced by RT [7, 8]. In this regard, it has been suggested that monitoring VL across sets could serve as a precise method of quantifying the so-called level of effort (i.e. repetitions performed with respect to the maximum number that can be completed) [5]. Indeed, recent studies have reported strong relationships between the VL experienced in a set and the percentage of performed repetitions with respect to the maximum number that can be completed in bench press and back-squat exercises with different loads [6, 9]. In one study [6], it was observed that the percentage of performed repetitions for a given magnitude of VL was very similar for all loads used, especially for those ranging between 50 and 70% 1RM, although the maximum number of repetitions completed against each relative load was significantly different. While this implies that VL should indeed be used to monitor and modify RT volume, it is important to acknowledge that the actual number of repetitions performed (i.e. volume of work) until reaching different VL thresholds was comparatively neglected as a consideration.

Many longitudinal studies compared the effectiveness of different VL thresholds on muscle strength, hypertrophy, and endurance as well as performance of athletic tasks. While the researchers generally concluded that the selected VL threshold can modulate these adaptations to training in a dose–response fashion [10, 11], only two studies to date matched the training volume between different VL thresholds [12, 13]. More specifically, when the training volume was matched between different VL thresholds, researchers reported no significant differences in the effectiveness of lower (i.e. 10 and 15%) and higher (20 and 30%) VL thresholds at inducing muscle strength and hypertrophy adaptations as well as improving performance of athletic tasks [12, 13]. This questions both the idea that the amount of VL allowed during a set—and not the volume of work completed—is what matters the most for inducing neuromuscular adaptations, and the usefulness of VL thresholds for prescribing RT volume. While the drawbacks of traditional methods of prescribing RT volume are often evidenced by the inter-individual variability in how many repetitions one can do until failure in each exercise and load, the VL method may suffer from the same problem. Furthermore, the relationship between VL and the number of repetitions performed might not be linear for all individuals. Specifically, some might perform multiple repetitions within a given VL threshold further contributing to the variability in the amount of work across individuals. In addition, it is likely that not all individuals can reach the same VL thresholds, especially higher ones (e.g. 50% VL), due to their characteristics, training status, and history. Although plausible, this is purely speculative as it has not been thoroughly examined to date.

Previously, researchers have reported that sex, training status (i.e. relative strength levels), and history should all be considered when designing a RT program and monitoring changes in neuromuscular function [4, 14, 15]. Thus, it can be hypothesised that these factors could also affect the individual responses to different VL thresholds. Personality traits could be another influential factor when it comes to implementing VL thresholds in practice. For instance, emotional stability and conscientiousness have been associated with fatigue tolerance [16, 17]. Given that VL thresholds are used to control intra-set fatigue and prescribe training volume, it may be that individuals with distinct personality traits might respond differently to the same VL threshold, potentially leading to divergent long-term neuromuscular adaptations. Finally, the stability of the amount of work completed after reaching different VL thresholds has not been thoroughly examined despite this method being widely used in practice. For example, knowing whether an individual who performs a given number of repetitions before reaching a given VL threshold in one training session will then perform the same number of repetitions before reaching that VL threshold a few days later, is critically important.

In light of these considerations and scarcity in the literature, an examination of the variability associated with different VL thresholds, and the influential factors associated with it is clearly needed. Therefore, the purpose of this study was to quantify the potential variability in the volume of work completed after reaching different VL thresholds and determine the effects of sex, training status and history, as well as personality traits on the reliability and magnitude of the amount of work completed after reaching different VL thresholds using different loads in the back-squat exercise. Based on the findings from previous longitudinal studies on the topic that matched VL threshold for training volume [12, 13], it was hypothesised that there would be notable variability in the volume of work completed across VL thresholds, which could at least partially be explained by some of the above-mentioned factors.

## Methods

### Design

This cross-sectional, test–retest study is part of a larger project investigating the reliability of different velocity-based RT monitoring and prescription methodologies. Participants reported to the laboratory on four occasions with 48–72 h of rest between the sessions. After familiarisation with the free-weight back-squat movement, all the equipment and instruments, instructions to move the barbell up as fast as they can, and feedback on the screen indicating the velocity of the barbell, participants performed an incremental loading (i.e. 1RM) test in the back-squat exercise. In the next two sessions, participants completed repetitions to failure (RTF) tests in the same exercise with 90, 80, and 70% of their established 1RM. Importantly, each participant performed all sessions at the same time of the day (± 1 h) to control for diurnal variation, and they were instructed to keep their habitual hydration, nutrition, and caffeine practices before every session the same (i.e. they were instructed to note what they have done before the first visit and then keep those practices for the remainder of the study). In addition, the same researchers were always present throughout all testing sessions for each participant and laboratory environment was kept the same (e.g. temperature and humidity) to avoid potential effects of these factors on performance throughout testing sessions.

### Participants

Fifty-one strength-trained people (15 females and 36 males; 18 to 40 years of age) participated in this study. The participants were recruited via word of mouth, online (social media), and physical flyers (e.g. university campuses and fitness centres) advertising the details of the study. Three male participants withdrew from the study due to injuries during their work or recreational sporting activity not related to the study, whereas two male participants dropped out of the study due to private reasons after completing one and three experimental sessions. Participants without a full data set were not analysed since the primary aim was to determine the reliability of the number of repetitions performed until reaching different VL. Therefore, the final number of participants included in the analysis was 46 (15 females and 31 males). The relative strength—expressed as 1RM to body mass ratio—in the free-weight back-squat exercise was 1.25 ± 0.30 and 1.79 ± 0.35 for females and males, respectively. To participate, participants had to confirm they (1) were willing to abstain from any additional lower-body training during the study; (2) were not currently taking medication that would alter metabolic or cardiovascular functions; (3) had no musculoskeletal limitations; (4) were not currently using anabolic steroids or had a history of use; and (5) had at least 6 months of RT experience training at least 2x/week including the back-squat exercise at least 1x/week, with no longer than 2 weeks in a row off training during that period. Importantly, all participants demonstrated technical competence in the free-weight back squat as confirmed by the visual inspection (e.g. back posture, coordination patterns of the hip, knee, and ankle, squatting depth) of the primary researcher during the familiarisation session. Each participant gave written informed consent before starting the study. The protocol of this study was in accordance with the ethical requirements of the University Ethics Committee (approval number: 20/55), and The Code of Ethics of the World Medical Association.

### Familiarisation Session

Upon arriving to the laboratory, participants first completed a questionnaire to better understand their training history and usual practices when it comes to RT (Additional file 1). Next, participants’ body mass and height were recorded using an electronic column scale and a wall-mounted stadiometer (Seca Ltd., Hamburg, Germany). Participants then completed a standardised warm-up consisting of cycling at 100 rpm for 5 min, dynamic stretching for 2 min, 10 bodyweight lunges and squats, as well as 10 barbell squats. Thereafter, participants were familiarised with the instruction to lift the barbell up as fast as they can (during the concentric muscle action), feedback on the screen indicating velocity of the barbell, and the instruction to have at least a momentary pause at the top of the movement not lasting longer than 2 s between repetitions with feet maintaining contact with the floor (i.e. no jumping or lifting of the heels) at all times. To ensure familiarity with these instructions and general conditions in which they were required to perform 1RM and RTF tests, participants then completed 3 repetitions at 20, 40, and 60% of their estimated 1RM and then 10 repetitions at 60% of their estimated 1RM. During these repetitions, participants were instructed to lift the barbell up as fast as they can and avoid pausing more than 2 s at the top of the movement (i.e. standing phase of the squat) while also receiving visual feedback indicating the velocity of the barbell. At the end of each session, all participants understood and felt comfortable with these conditions and performed at least two sets with consistent repetition velocities (i.e. all repetitions with a given load were performed with a small deviation in velocity ± 0.02 m/s). Participants were also asked to provide logs of their most recent (and heaviest) back-squat sessions and to conservatively estimate their 1RM. This information was used to establish warm-up loads for the upcoming 1RM sessions.

### One Repetition Maximum Session: Day 2

Prior to the 1RM assessments, participants completed the same standardised warm-up as in the familiarisation session. The 1RM assessment was performed using a 20-kg barbell (Rogue, Columbus, Ohio, USA) and calibrated weight plates (Eleiko; Halmstad, Sweden, EU). The 1RM protocol consisted of 3 repetitions each at 20, 40, and 60%, and 1 repetition at 80, and 90% of an estimated 1RM, followed by 1RM attempts. After each successful attempt, the load was increased in consultation with the participant from 1 to 12.5 kg until no further weight could be lifted or until movement technique was compromised. In addition, a maximum of five 1RM attempts were allowed for each participant. Three and four minutes of passive rest were provided between each submaximal set and 1RM attempts, respectively. To ensure natural squatting patterns, each participant adopted a shoulder width stance and used a self-regulated eccentric velocity; immediately upon reaching the bottom of their squat, participants were instructed to perform the concentric (upward) portion of each repetition as fast as possible. Strong verbal encouragement and visual feedback were provided throughout all trials. Participants were required to reach a depth of the squat at which the top of the thighs was at least parallel to the floor, as determined by the investigators and a camera positioned perpendicularly to the participant, for a repetition to be considered successful.

### Repetitions to Failure Sessions: Days 3 and 4

The same standardised warm-up as in the familiarisation and 1RM sessions was performed during the RTF sessions. Thereafter, participants completed four sets of 10, 5, 3, and 1 repetition of the free-weight back-squat exercise against 30, 50, 70, and 90% of the 90% of their established 1RM (i.e. the heaviest load to be lifted that day). They were provided with 3 min of rest between warm-up sets and 4 min between the last warm-up set and the first set to failure. After the general and specific warm-up, participants performed three sets with 90, 80, and 70% 1RM, respectively, to failure with 10 min of rest between sets. Since the excessive fatigue from performing a high number of repetitions during RTF with lower loads (i.e. 70% 1RM) could have compromised the number of repetitions performed during subsequent RTF sets with greater loads (i.e. 80 and 90% of 1RM), the loads were not tested in a randomised fashion. Instead, participants always performed RTF with the highest load (i.e. 90% 1RM) first while the last RTF set was always performed with the lowest load (i.e. 70% 1RM). With regard to the exercise execution (including lifting instructions, encouragement, and visual feedback), the same conditions applied as during the 1RM session. The rest between two successive RTF sessions was 72 h.

### Data Acquisition

The questionnaire pertaining to the training history and practices of participants is described in Additional file 1. Briefly, the questions were related to the (1) number of repetitions participants perform during their own training, on average; (2) the intensity of load at which participants train, on average; (3) the number of repetitions left in reserve after completing their training sets, on average; and (4) the experience they have with RT, in years. These questions were multiple-choice; hence, the responses were treated as categorical. After inspecting frequencies of responses for each category (both overall and within levels of outcome variables used in models), these variables were recoded by merging some response options to avoid having < 5 responses in more than 20% of cells/categories [18]. In particular, the number of repetitions performed was transformed into a categorical variable with three levels (1–5, 5–8, > 8), as well as was the intensity of load (< 70, 70–80, 80–90), whereas the number of repetitions left in reserve and the experience with RT were transformed into categorical variables with two levels (0–2 and 2–4; ≤ 3 and > 3, respectively).

Mean velocity of all the repetitions was recorded using the GymAware (manufacturer) linear position transducer (LPT). The GymAware is a commercially available LPT consisting of a power tool, made up of a steel cable that is wound on a cylindrical spool coupled to the shaft of an optical encoder. The power tool units were placed on both sides of the barbell perpendicular to the position between the hands and the loaded barbell sleeves, according to the manufacturer’s instructions. The end of the cable was vertically attached to the barbell using a Velcro strap. Its placement on the barbell was standardised throughout the study, and the cable coming out of the power tool was virtually vertical with respect to the barbell at the beginning of each set [19]. The LPT of the current study measures the total displacement of its cable in response to changes in the barbell position and incorporates an angle sensor that accounts for motion in the horizontal direction during predominantly vertical displacement measurements. The software later accounts for the total distance and angle, and using basic trigonometry, provides a resultant vertical displacement. Instantaneous velocity was determined as the change in barbell position with respect to time, which is also provided by the LPT’s software. More detailed specifications of this LPT, as well as its reliability and validity data, were previously reported [19–21]. Data obtained from the LPT were transmitted via Bluetooth to a tablet (iPad, Apple Inc., California, USA) using the GymAware v2.8.0 app. The LPT attached to the right side of the barbell was connected to the TV and provided visual feedback indicating the velocity of the barbell after each repetition. The data from this LPT were used for the analysis. Finally, to avoid any data loss due to issues with online clouds or the internet connection, the mean velocity of all repetitions was manually recorded and organised in the Microsoft Excel spreadsheet (Microsoft Corporation, Redmond, Washington, USA) during each session. To ensure the consistency and accuracy of this procedure, the same two researchers handled this task throughout the study.

The 50-item International Personality Item Pool (IPIP) Big Five Personality Inventory was used as a validated form of human personality assessment [22, 23]. The inventory contained 50 questions, 10 for each of the Big Five personality dimensions (Agreeableness, Conscientiousness, Extraversion, Neuroticism, and Openness). The IPIP items were administered with a 5-point, Likert-type scale ranging from 1 (*very inaccurate*) to 5 (*very accurate*) as in the original instrument using Qualtrics survey-building software. The scores for each item were averaged to obtain mean scores across personality dimensions. For the purposes of the present study, only Conscientiousness and Neuroticism (or Emotional stability) were retained for the analysis.

### Statistical Analysis

**Fig. 1:**
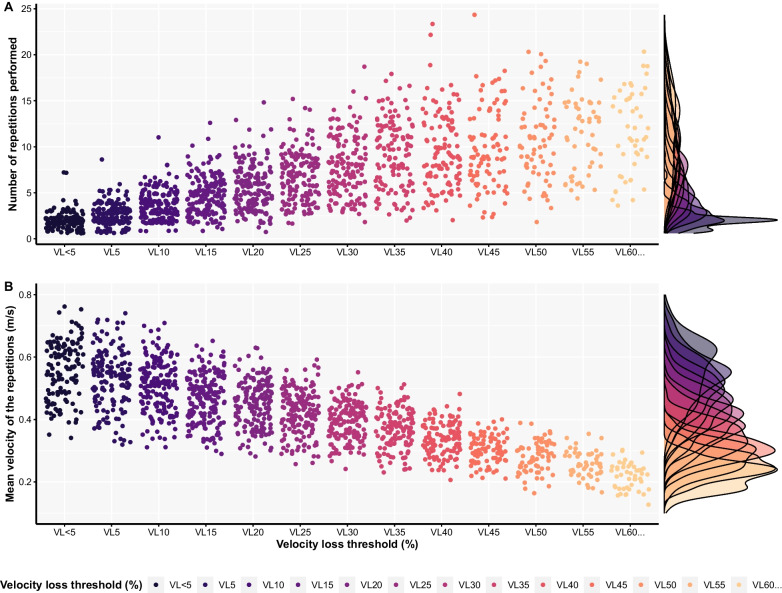
Visual representation of the number of repetitions (**A**) and velocity of repetitions associated with the first instance when a given velocity loss threshold was exceeded (**B**) performed across velocity loss thresholds, loads, and both testing sessions for all individuals

Individual repetitions and repetitions’ velocity across VL thresholds are visualised in Fig. 1 and Additional file 2. The agreement across days between the (1) number of repetitions performed until reaching a given VL threshold (i.e. from < 5% to 60%> in 5% increments) and (2) velocity associated with the repetitions when a given threshold was first exceeded was examined. For this purpose, the modified true value varies method of the Bland–Altman analysis for multiple observations per participant [24] was used for each load separately. The bias and associated 95% limits of agreement (LoA) were evaluated and interpreted in the context of an *apriori* specified equivalent margin of ± 2 repetitions and ± 0.06 m/s, for repetitions and velocity of the repetitions, respectively. These criteria were selected since a more than two repetitions difference would not represent an improvement over more traditional RT prescription methods such as those using the rating of perceived exertion [25, 26], whereas the smallest detectable change in velocity according to the load-velocity relationship in the free-weight back squat was reported as 0.06 m/s [27]. The confidence intervals for LoA were calculated according to the method of variance estimates recovery (MOVER) method, which considers the repeated measurements taken [28]. In addition, for LoA, equivalence tests were also performed via two one-sided tests (TOSTs). The TOST procedure was performed with an α-value of 0.1 and a 1─2α confidence interval. The null hypothesis of TOST was that the two values were not equivalent. If the 1–2α confidence interval was completely contained within the ± equivalent margin, the null hypothesis was rejected, and the two datasets (i.e. number of repetitions or velocities associated with a given threshold from day 1 and day 2) were considered equivalent [29]. All assumptions of the Bland–Altman analysis were satisfied.

Linear mixed-effects models with the Gaussian conditional distribution and identity link function were used to examine what affected the number of repetitions performed until reaching a given VL threshold and the velocity associated with the repetitions when a given VL threshold was exceeded for the first time (i.e. outcome measures). For this purpose, the load (3 levels), training experience (2 levels) and practices (2 or 3 levels), relative strength and conscientiousness and emotional stability were all considered as fixed effects.

The same approach as described above was also used to investigate (1) factors affecting the probability of not reaching the same VL thresholds in both days among participants; (2) factors explaining whether a participant would perform multiple repetitions^1^ within a single VL threshold; and (3) factors affecting the probability of reaching a 50% VL in a set (i.e. the ability to endure low velocity repetitions). However, for all three analyses, generalised linear mixed effects models were used. A binomial conditional distribution was specified with a logit link function to predict the odds of reaching the same VL on both days, performing multiple repetitions within a single VL threshold, and being able to endure a 50% VL in a set.

For all models, participants (*n* = 46) and VL thresholds (*n* = 13) were treated as random effects giving rise to cross-classified random effects models, while their interaction was also introduced in the models when their addition did not result in a convergence error and when it improved the model fit. This was done to control for repeated measurements, and the general variation between participants, VL thresholds and participants’ performance within certain VL thresholds. Since both fixed and random effects were used, restricted maximum likelihood estimation was used for evaluation of the linear mixed effect models, whereas maximum likelihood, with Laplace approximation, estimation was used for generalised linear mixed-effects models. The contribution of both fixed and random effects to the explanatory power of any of the explored models was examined using a likelihood ratio test, deviance statistic, and Akaike information criterion score, before selecting the final model to obtain the best-fit model while maintaining model parsimony. Importantly, the reduction of the model structure was always theoretically motivated and was done as a last resort. Statistical significance of fixed effects was examined by t-tests based on the Satterthwaite approximation or Wald *Z*-tests for linear mixed effects and generalised mixed-effects models, respectively. For linear mixed-effects models, estimated marginal means and 95% confidence intervals (95% CI) were calculated and presented, whereas for generalised mixed-effects models odds ratios as well as predicted probabilities with associated 95% CI were evaluated and presented to aid interpretation of the findings. For categorical predictors with more than 2 levels, post hoc tests were performed with Holm–Bonferroni correction. More details on models’ specification and diagnostics, as well as software packages used, can be found in the Additional file 3.

To explore whether performing a higher number of repetitions until a given VL threshold results in a higher total number of repetitions performed in a set, Pearson product–moment correlations were calculated for 10, 20, 30, and 40% VL across 70, 80, and 90% of 1RM. Prior to this, all data were examined and confirmed for normality via graphical inspection and the indicator value range for skewness and kurtosis [18].

All statistical analyses were conducted using the R language and environment for statistical computing (version 4.2.0, The R foundation for Statistical Computing, Vienna, Austria). Custom-written R script and associated dataset are available at the Open Science Framework repository (https://osf.io/3cnw8/).

## Results

**Fig. 2:**
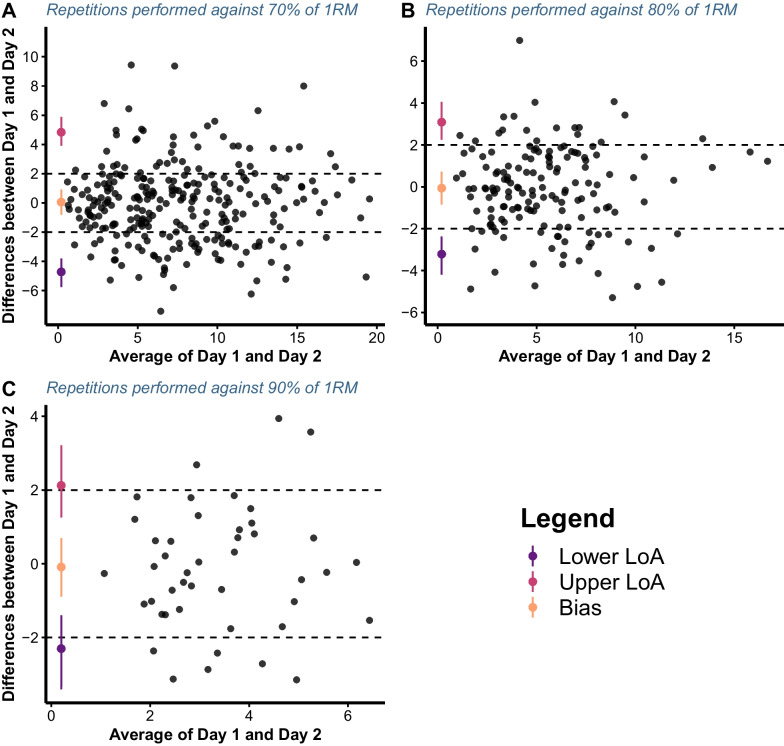
Bland–Altman plots illustrating the agreement between the number of repetitions performed until reaching a given velocity loss threshold on two testing sessions for 70 (**A**), 80 (**B**), and 90% of 1RM load (**C**). Dashed lines represent equivalent margin of ± 2 repetitions, whereas LoA represents limits of agreement

**Fig. 3:**
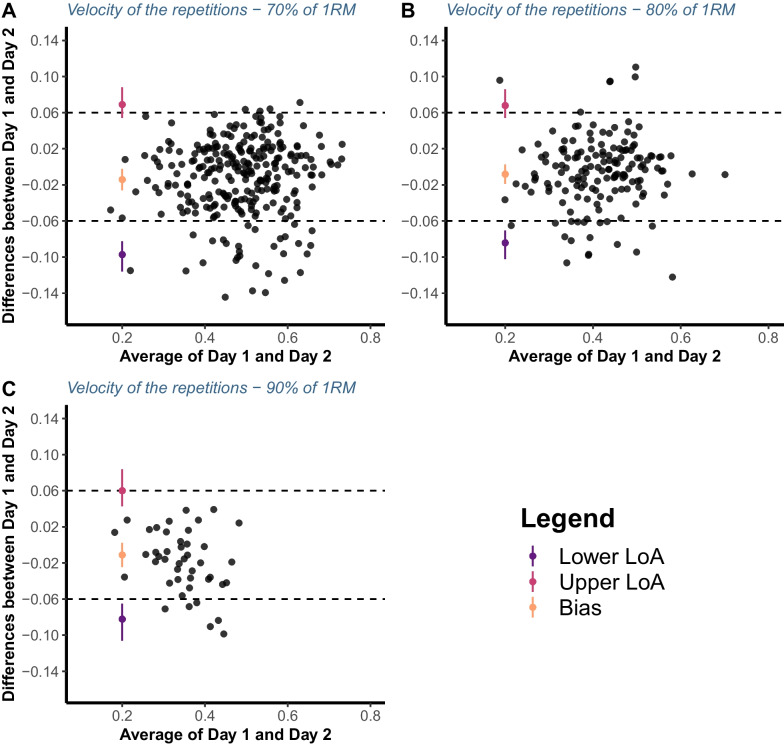
Bland–Altman plots illustrating the agreement between the repetitions velocity associated with the first instance a given velocity loss threshold was exceeded on two testing sessions for 70 (**A**), 80 (**B**), and 90% of 1RM load (**C**). Dashed lines represent equivalent margin of ± 0.06 m/s, whereas LoA represents limits of agreement

The null hypothesis for the equivalence of the number of repetitions performed until reaching a given VL threshold on day 1 and day 2 was not rejected for any of the examined loads since the 1–2α confidence interval of LoA either crossed or were completely outside the ± equivalent margin of 2 repetitions (Fig. 2). Similarly, the null hypothesis for the equivalence of the mean velocity of repetitions associated with the first instance when a given VL threshold was exceeded on day 1 and day 2 was also not rejected for any of the loads examined due to 1–2α confidence intervals of LoA being completely outside the ± 0.06 m/s margin of equivalence (Fig. 3).

**Fig. 4:**
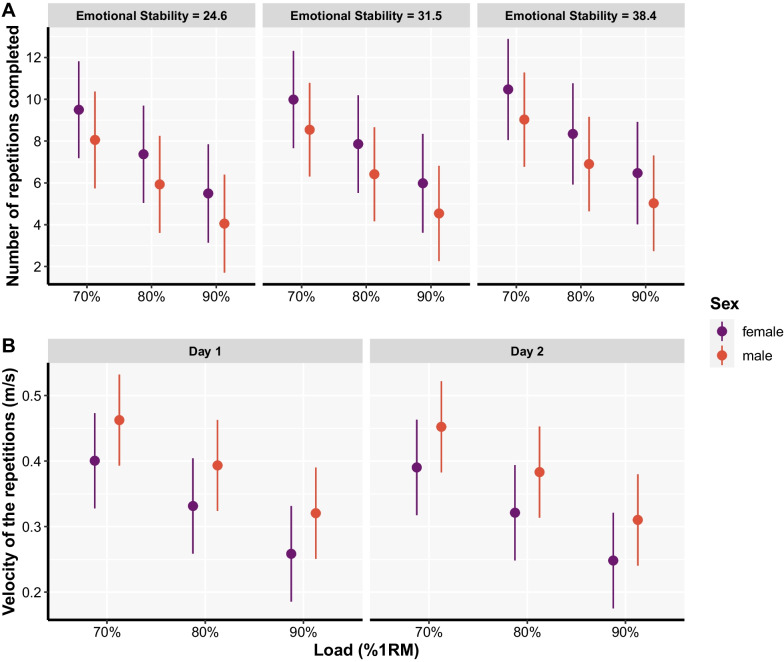
Estimated marginal means with 95% confidence intervals for the number of repetitions completed across velocity loss thresholds (**A**) and velocity of repetitions associated with the first instance a given velocity loss threshold was exceeded (**B**). For model estimates, please refer to Tables 1 and 2, and for pairwise comparisons related to the loading condition please see Additional file 4

The number of repetitions performed until reaching a given VL threshold was affected by sex, load, and emotional stability (Table 1; Fig. 4). Females performed more repetitions than males (*p* = 0.011), number of repetitions performed linearly decreased as the load increased (*p* < 0.001; Additional file), and number of repetitions performed was higher among people with greater emotional stability. The mean velocity of repetitions associated with a first instance when a given VL threshold was exceeded was affected by day, sex, and load (Table 2; Fig. 4). Males displayed greater mean velocities than females (*p* = 0.005), and mean velocity linearly decreased as the load increased (*p* < 0.001; Additional file 4), and mean velocity of repetitions was generally greater on day 1 compared to day 2 (*p* < 0.001).

**Table 1:** Factors affecting: (1) number of repetitions performed until reaching a given velocity loss threshold; (2) the velocity of the repetitions associated with the first instance when a given velocity loss threshold was exceeded

Predictors	N of repetitions until reaching velocity loss thresholds			Velocity when first exceeding velocity loss thresholds		
	Statistics			Statistics		
	Estimates	CI	*p*	Estimates	CI	*p*
(Intercept)	6.20	1.83 – 10.56	0.005	0.53	0.37 – 0.68	< 0.001
Day [Day 2]	−0.04	−0.26 – 0.17	0.683	−0.01	−0.01 – −0.01	< 0.001
Sex [male]	−1.44	−2.56 – −0.33	0.011	0.06	0.02 – 0.11	0.005
Load [80% 1RM]	−2.13	−2.39 – −1.86	< 0.001	−0.07	−0.07 – −0.07	< 0.001
Load [90% 1RM]	−4.00	−4.44 – −3.56	< 0.001	−0.14	−0.15 – −0.14	< 0.001
Emotional stability	0.07	0.01 – 0.13	0.027	−0.00	−0.00 – 0.00	0.190
Conscientiousness	−0.00	−0.07 – 0.07	0.925	−0.00	−0.00 – 0.00	0.898
Training experience [> 3 years]	−0.18	−1.05 – 0.70	0.692	−0.00	−0.04 – 0.03	0.872
Repetition practices [8–12]	−0.13	−1.08 – 0.82	0.789	−0.00	−0.04 – 0.04	0.935
Repetition practices [> 12]	0.26	−0.75 – 1.27	0.610	−0.00	−0.05 – 0.04	0.820
Relative strength (1RM/BM)	1.11	−0.18 – 2.39	0.091	−0.04	−0.09 – 0.01	0.083
Loads practices [70–80% 1RM]	–	–	–	0.00	−0.04 – 0.05	0.858
Load practices [> 80% 1RM]	–	–	–	−0.01	−0.06 – 0.04	0.631
Repetitions in reserve practices [> 2 RIR]	–	–	–	0.01	−0.03 – 0.04	0.693
*Random effects*						
*σ*^2^	2.80			0.00		
*τ*_00_ _ID:VL_	0.95			–		
*τ*_00_ _ID_	1.34			0.00		
*τ*_00_ _VL_	15.91			0.01		
ICC	0.87			0.96		
*N*_ID_	46			46		
*N*_VL_	13			13		
Observations	944			944		
Marginal *R*^2^/Conditional *R*^2^	0.095/0.879			0.131/0.965		

Reference groups were the following: Day [Day 1], Sex [female], Load [70% 1RM], Training experience [< 3 years], Repetitions practices [< 8], Load practices [< 70% 1RM], Repetitions in reserve practices [< 2 RIR]

*1RM* one repetition maximum, *BM* body mass, *RIR* repetitions in reserve, *ICC* interclass-correlation coefficient, *R*^*2*^ pseudo coefficient of determination, *CI* 95% confidence intervals, *p* p value

**Table 2:** Factors affecting whether participants: (1) reached the same velocity loss threshold in two consecutive days; (2) performed multiple repetitions within a single velocity loss threshold; (3) were able to experience 50% velocity loss within a training set

Predictors	Reaching the same velocity loss threshold on both days			Multiple repetitions within a single velocity loss threshold			Experiencing 50% velocity loss within a set		
	Statistics			Statistics			Statistics		
	Odds ratios	CI	*p*	Odds ratios	CI	*p*	Odds ratios	CI	*p*
(Intercept)	0.66	0.11–3.83	0.645	0.09	0.03–0.31	< 0.001	0.24	0.00–59.06	0.609
Sex [male]	1.16	0.69–1.95	0.582	0.45	0.31–0.66	< 0.001	0.52	0.09–3.10	0.477
Load [80% 1RM]	2.43	1.82–3.24	< 0.001	0.42	0.33–0.54	< 0.001	0.55	0.15–1.94	0.351
Load [90% 1RM]	8.10	5.44–12.07	< 0.001	0.10	0.07–0.16	< 0.001	0.03	0.01–0.19	< 0.001
Emotional stability	1.00	0.97–1.02	0.751	1.04	1.02–1.06	< 0.001	1.17	1.02–1.34	0.029
Conscientiousness	1.00	0.97–1.03	0.971	1.00	0.98–1.02	0.794	0.99	0.87–1.13	0.900
Training experience [> 3 years]	0.85	0.57–1.28	0.449	0.82	0.61–1.09	0.168	–	–	–
Repetition practices [8–12]	0.93	0.59–1.47	0.753	0.94	0.68–1.31	0.736	–	–	–
Repetition practices [> 12]	1.14	0.69–1.89	0.604	1.78	1.25–2.55	0.002	–	–	–
Relative strength (1RM/BM)	1.30	0.73–2.33	0.376	2.23	1.47–3.40	< 0.001	–	–	–
Load practices [70–80% 1RM]	0.94	0.56–1.59	0.824	0.81	0.56–1.18	0.273	–	–	–
Load practices [> 80% 1RM]	0.78	0.43–1.40	0.401	1.38	0.91–2.10	0.129	–	–	–
Repetitions in reserve practices [> 2 RIR]	0.93	0.62–1.40	0.734	1.67	1.25–2.23	0.001	0.28	0.05–1.69	0.165
*Random effects*									
*σ*^2^	3.29			3.29			3.29		
*τ*_00_ _ID_	0.12			0.00			3.93		
*τ*_00_ _VL_	0.38			0.19			–		
ICC	0.13			0.05			0.54		
*N* _VL_	13			13			–		
*N* _ID_	46			46			46		
Observations	1121			1593			138		
Marginal *R*^2^/Conditional *R*^2^	0.155/0.267			0.199/0.242			0.333/0.696		

Reference groups were the following: Load [70% 1RM], Sex [female], Training experience [< 3 years], Loads practices [< 70% 1RM], Repetitions practices [< 8], Repetitions in reserve practices [< 2 RIR]

*1RM* one repetition maximum, *BM* body mass, *RIR* repetitions in reserve, *ICC* interclass-correlation coefficient, *R*^2^ pseudo coefficient of determination, *CI* 95% confidence intervals, *p* p value

**Fig. 5:**
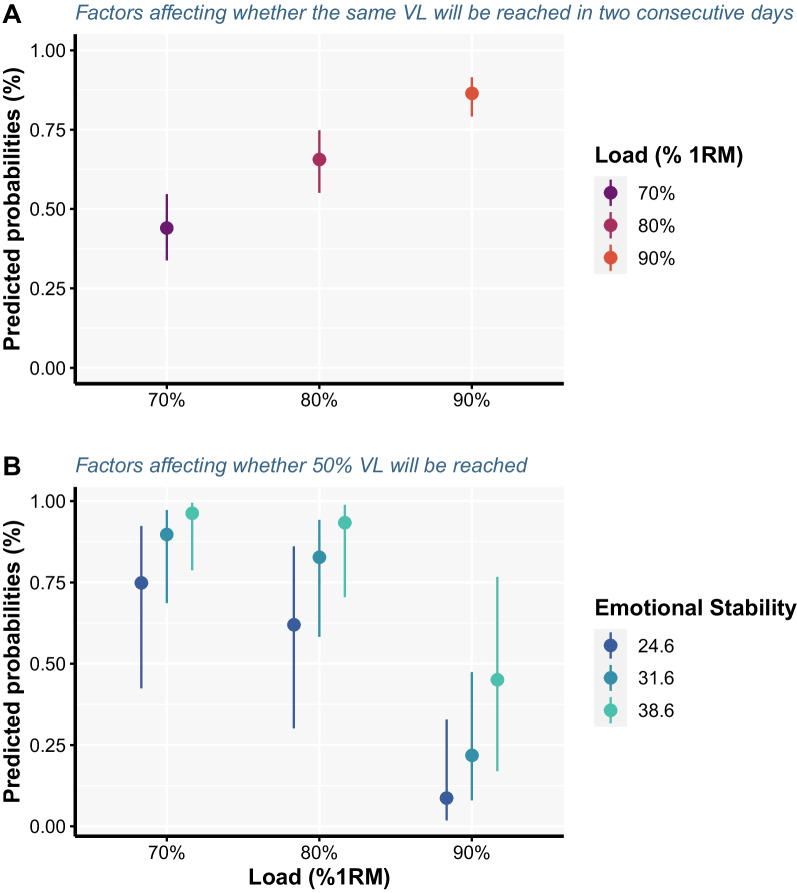
Predicted probabilities with 95% confidence intervals for reaching the same velocity loss threshold on two testing sessions (**A**) and for reaching a 50% velocity loss in a set (**B**). For model estimates, please refer to Tables 1 and 2, and for pairwise comparisons related to the loading condition please see Additional file 4

The model examining factors affecting the probability of not reaching the same VL thresholds on both days among participants revealed only load was an influential factor, with the probability of not reaching the same VL threshold across days linearly decreasing as the load increased (*p* < 0.001; Fig. 5; Additional file 4).

**Fig. 6:**
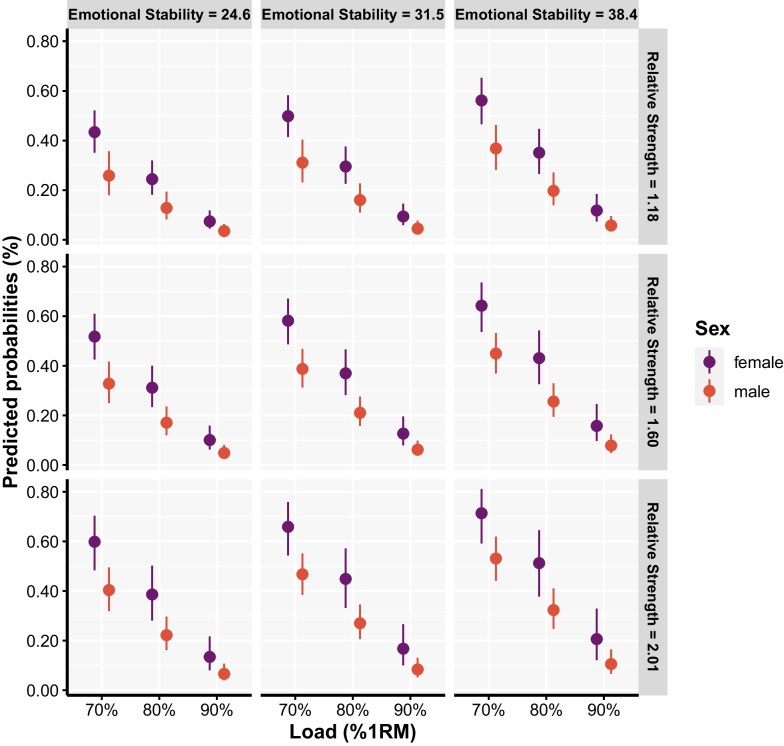
Predicted probabilities with 95% confidence intervals for performing multiple repetitions within a single velocity loss threshold. Columns make distinction between different levels of emotional stability, whereas rows make a distinction between different strength levels of the participants. For model estimates, please refer to Tables 1 and 2, and for pairwise comparisons related to the loading condition, please see Additional file 4

The model examining factors affecting the probability of doing multiple repetitions within a given VL threshold revealed the load, sex, emotional stability, strength level, repetitions in reserve training history, and number of repetitions typically performed per set were influential factors (Table 2; Fig. 6). Females had a greater probability of doing multiple repetitions within a single VL threshold than males (*p* < 0.001), the probability of doing multiple repetitions linearly increased as load decreased (*p* < 0.001) and was greater for stronger (*p* < 0.001), more emotionally stable participants (*p* < 0.001), and among participants who generally performed a higher number of repetitions (*p* = 0.002) and left 2–4 repetitions left in reserve during their own training (*p* < 0.001).

The model examining factors affecting the probability of reaching 50% VL in a set (i.e. the ability to endure low velocity repetitions) revealed only load and emotional stability as influential factors with probability of reaching 50% VL in a set being higher for 70 (*p* < 0.001) and 80% (*p* < 0.001) compared to 90% of 1RM, and for participants who scored higher on emotional stability (*p* = 0.029).

The relationship between the number of repetitions performed until reaching 10, 20, 30, and 40% VL and the total number of repetitions completed in a set was generally stronger for higher (i.e. 30 and 40% VL) compared to lower VL thresholds, regardless of the load (i.e. 70, 80, or 90% of 1RM) used (Additional file 5).

## Discussion

To our knowledge, this is the first study that comprehensively examined the reliability and magnitude of work completed across VL thresholds during RT while also exploring the effects of several factors potentially influencing the use of VL thresholds in practice. The main findings of this study were (1) the agreement of the number of repetitions performed across VL thresholds between two testing sessions was not acceptable, regardless of the load used; (2) the agreement of the velocities associated with the first instance when a given VL threshold was exceeded between two testing sessions was not acceptable, regardless of the load used; (3) the number of repetitions performed across VL thresholds was affected by sex, load, and emotional stability, whereas the magnitude of the velocity associated with the first instance a given VL threshold was exceeded was affected by sex, testing session, and load; (4) whether the same VL threshold was reached by the same person in two consecutive days was affected by load, whereas whether someone would experience a 50% VL in a set depended upon the load used and emotional stability of the person; and (5) whether someone would perform multiple repetitions within a given VL threshold was affected by sex, load, training history, strength, and emotional stability. Considering the above, it can be posited that (1) VL thresholds cannot be used to reliably control the amount of work completed during RT, but rather as a general variable quantifying the amount of fatigue experienced by an individual and (2) VL thresholds should be highly individualised when used in practice since load, sex, training status, history, and psychological traits can all influence, at least to some extent, the variability in responses to different VL thresholds.

The agreement of the actual volume of work completed until reaching different VL thresholds between two consecutive testing sessions (72 h apart) was not acceptable since the limits of agreement constantly sat outside the ± 2 repetitions margin, regardless of the load used. For instance, with 70% of 1RM, the upper CI of the upper LoA (estimate = 5.47) and lower CI of the lower LoA (estimate = −5.36) were higher and lower than 5 repetitions, respectively. This effectively means that, for the same threshold, individuals can be expected to perform anywhere between less than 5 and more than 5 repetitions on two consecutive testing sessions in a controlled environment (i.e. laboratory settings). While the extent to which this difference in work would affect longitudinal adaptations is not currently quantified, it is likely that the effect is profound. This contention is supported by the discrepancy in the literature regarding the effectiveness of various VL thresholds for inducing training adaptations. For instance, lower VL thresholds were equally effective and more beneficial for muscle strength and power adaptations, whereas higher VL thresholds were superior for muscle hypertrophy compared to lower ones [30]. However, when the volume of work was matched between different VL threshold groups, no differences were observed in muscle strength, hypertrophy or even gains in performance of athletic tasks [12, 13]. In addition, individuals might not reach the same VL threshold on two consecutive training sessions, as found in the present study (Fig. 5b), which compromises the ability of this method to control RT volume. This would only be exacerbated outside of controlled laboratory environments due to the complex interplay of different levels of fatigue, motivation, prior injury, and general readiness to train across individuals in common practical settings. Finally, the agreement of the velocities associated with the first instance when a given VL threshold was exceeded between two testing sessions was also not acceptable, regardless of the load used. Therefore, VL thresholds do not seem to be a reliable option for monitoring and prescribing RT volume.

The number of repetitions performed across VL thresholds was affected by sex, load, and emotional stability, whereas the velocity associated with the first instance a given VL threshold was exceeded was affected by sex, load, and day. In this regard, both the number of repetitions and repetition velocity decreased as the load increased across VL thresholds. Although this finding may seem logical, it implies that the same amount of work cannot be completed just by selecting a given VL threshold, as the work completed is also load dependent. Thus, monitoring and performance evaluation (e.g. pre- to post-training cycle) should be done across the loading spectrum, while comparing performances for each load individually. Furthermore, regardless of the load used, females completed more repetitions than males across VL thresholds, while males performed repetitions at higher velocities. These findings align with the reported physiological and neuromuscular differences, often resulting in differences in exercise performance and recovery, between men and women [31]. Specifically, on average, men are stronger, and women are less fatigable; able to sustain force at the same relative intensity for a longer period [32, 33]. These sex differences in strength and fatigability were previously attributed to variation in muscle phenotype [34] insofar that women have smaller muscle fibres than men [34] and a higher proportion of type I fibres relative to type II [35], with greater muscle capillarisation [36], blood flow during exercise [37], and with distinct glycolytic and oxidative capacities [38, 39]. While relative strength did not influence the number of repetitions performed or repetition velocity across VL thresholds in the present study, sex differences in fatiguability could explain why females did more work, on average, compared to males across VL thresholds, and sex differences in the proportion of type I fibres relative to type II fibres, could explain why males performed repetitions at higher velocities, on average, across VL thresholds.

In the present study, individuals with higher levels of emotional stability tended to perform more repetitions across VL thresholds, on average. In general, high scorers on neuroticism—the trait on the opposite spectrum of emotional stability—tend towards heightened perception of fatigue symptoms [17]. Indeed, elevated neuroticism, by definition, is related to more frequent and intense experience of negative emotional states, and elevated neuroticism is positively associated with fatigue [16, 17]. Furthermore, individuals with higher levels of neuroticism feel exhausted more frequently and report more severe fatigue than those with lower neuroticism [40]. It may be that individuals higher in emotional stability have in fact stronger fatigue tolerance (rather than propensity to experience less fatigue), thus enabling them to complete a greater amount of work across VL thresholds. Importantly, conscientiousness did not affect the number of repetitions performed or repetition velocity across VL thresholds in the present study, although this personality trait has been previously linked with fatigue [16]. Conscientiousness generally describes people who are task oriented. Since all participants who completed all the procedures were verbally encouraged and acknowledged for their efforts during the study, it may be that this was enough for people of all levels of conscientiousness to feel as though the task was successfully completed, thus preventing any influential effects of this personality trait that might have occurred if the experimental situation was less structured without clear instructions, encouragement, and acknowledgments for completing the task (i.e. the testing session). The amount of VL experienced during RT has been repeatedly shown to be a useful, non-invasive indicator of the acute metabolic stress, hormonal response, and mechanical fatigue during RT and as such could be used to quantify fatigue induced by the training set [7, 8]. However, it seems that this monitoring practice could be drastically improved by considering individual trainee characteristics and training conditions since sex, the choice of load, and emotional stability all affected responses to different VL thresholds during RT.

There are several considerations when implementing velocity-based approaches to RT. For instance, the repetition–velocity relationship might not be linear for all individuals [41], and sets can be terminated after one or two repetitions exceed a predetermined threshold [42]. It seems logical that these considerations also apply to using VL thresholds during RT. It may be that whether individuals (1) can perform multiple repetitions within a single VL threshold and (2) have the capacity to experience a 50% VL in a set are additional factors worth considering when implementing VL thresholds in practice, since both will likely affect the repetition–velocity relationship, as well as when sets are terminated. Indeed, whether individuals can do multiple repetitions within a single VL threshold is further influenced by sex, load, strength, emotional stability, and prior training practices. In this regard, females, stronger individuals, individuals higher in emotional stability, and those who generally perform higher repetitions in training (> 12) and leave 2–4 repetitions in reserve are more likely to perform multiple repetitions within a single VL threshold. The effects of sex may be attributed to differences in fatiguability and muscle phenotype between males and females, whereas those with higher emotional stability had greater fatigue tolerance, allowing them to complete more work within the same VL threshold. It is not surprising that stronger individuals, and those who perform higher number of repetitions during their own training were more likely to perform multiple repetitions within a single VL threshold. Namely, stronger individuals would generally possess greater neural drive [43], myofibrillar cross-sectional area [44], and superior intermuscular coordination [45], all likely enhancing the ability to complete more work. In addition, generally performing a high number of repetitions during RT increased the odds of doing multiple repetitions within a single VL threshold likely due to these participants having greater muscle endurance (i.e. training specificity principle). Interestingly, those who usually left 2–4, compared to 0–2 reps in reserve in their own training, were more likely to perform multiple repetitions within a single VL threshold. Furthermore, only load and emotional stability explained the ability of the individuals to experience a 50% VL during RT. Indeed, experiencing 50% VL with higher loads is unlikely, given that repetition velocity sharply decreases as the load increases, thus not giving the opportunity to reach high VL. However, individuals higher in emotional stability were more likely to experience high VL during RT, probably due to their ability to cope with fatigue, as previously alluded to. At least some of these findings could also be explained by the fact that individuals have their own “pattern” of experiencing VL (Additional file 6). For instance, some people experience a gradual decline in velocity as the number of repetitions increase, whereas others maintain high velocity in the beginning and then experience a sudden drop in velocity, while others experience an early drop in velocity and then maintain velocity near the end of a set. The exploratory correlations between the number of repetitions performed until reaching 10, 20, 30, and 40% VL and the maximum number of repetitions completed in a set across loads support the concept that individuals have unique VL patterns. If there was indeed a general pattern of VL, one would assume very high correlations (*r* > 0.9) between the number of repetitions performed until reaching any of the VL thresholds and the maximum number of repetitions performed in that set (until failure). However, this was not the case, especially not with the loads (e.g. 70–80% 1RM) and VL thresholds (0–20%) typically used to optimise neuromuscular adaptations during RT, as the correlations never surpassed *r* = 0.63 (Additional file 5). While this insight is entirely exploratory, it seems reasonable to hypothesise that individuals possess their own patterns of VL, which limits the generalisability of specific VL threshold stimuli across individuals. Considering all the above, it seems prudent to consider the choice of load and individual trainee characteristics (i.e. training status, history, and psychological traits) when implementing VL thresholds in practice as each affects the variability of responses.

The present study took a unique approach to examine a range of factors related to the use of VL thresholds during RT, and there are several considerations when interpreting the data. Firstly, the reliability and magnitude of the work completed until reaching different VL thresholds may not necessarily transfer to other exercises. However, the effects of trainee characteristics are likely applicable to a variety of exercises. Secondly, while attempts were made to represent a broad range of participants (e.g. training experience, strength levels, sex, etc.), these findings may not generalise to sedentary populations since the participants had at least 6 months of RT experience. This at least partially explains why training experience did not influence any of the outcomes in the present study. Thirdly, since not every set in the present study was performed in a completely fresh state, some residual fatigue may have affected performance in subsequent sets and perhaps between day agreement for the amount of work completed across VL thresholds. However, 72 h of rest between sessions and long rest intervals between sets, with participants performing sets with loads in a descending order should have ensured that the potential effects of fatigue were minimised. Additionally, the mean absolute difference in velocity between the two testing sessions for 70, 80, and 90% of 1RM was 0.05, 0.04, and 0.04 m/s, respectively, which is lower than the smallest detectable change in velocity reported by Banyard et al. [27] in the free-weight back-squat exercise. This datum further supports that the rest periods in this study were appropriate. Fourthly, while efforts were made to balance the numbers of female and male participants, the number of females in the present study was considerably lower compared to males (due to COVID-related issues with recruitment). However, the females had a wide range of strength levels, training experience, and different training practices, improving this sample’s generalisability. Nevertheless, future studies should aim to balance the number of male and female participants when making comparisons, where possible. Finally, the present study could not determine variability in metabolic, neuromuscular, and biomechanical responses to different VL thresholds. Therefore, future studies should quantify the extent that the demonstrated variability in the amount of work completed until reaching different VL thresholds affects the variability in acute metabolic, neuromuscular, and biomechanical responses to VL thresholds, as well as in longitudinal adaptations.

## Conclusions

The present study expands on the previous velocity-based RT literature while providing several novel findings. The results question the utility of using VL thresholds to prescribe RT volume as the agreement in the amount of work completed across two consecutive testing sessions was not acceptable. Furthermore, while monitoring VL during RT is often used as a non-invasive indicator of the acute metabolic stress, hormonal response, and mechanical fatigue during RT, findings of the present study suggest that VL monitoring practices could be further improved by considering sex, training status, history, and psychological traits of individuals due to their effects on the variability in responses to different VL thresholds. In addition, it is also worth considering individual patterns of experiencing VL in a set as trainee’s characteristics (e.g. emotional stability) and training conditions (i.e. the choice of load) can determine the relationship between VL and the number of repetitions completed. Therefore, using the same VL thresholds for all individuals, while assuming generalisability of the stimuli applied, would likely lead to variable acute physiological responses to RT and divergent neuromuscular adaptations over the long term.

### Supplementary Information

**Additional file 1:** Details on the questions related to training experience and practices.

**Additional file 2: Figure S1.** Individual repetitions and repetitions’ velocity across velocity loss (VL) thresholds.

**Additional file 3:** Details on model specification and diagnostics.

**Additional file 4:** Pairwise comparisons for categorical variables with more than 2 levels for all outcomes of interest.

**Additional file 5: Figure S2.** Relationships between the number of repetitions performed until reaching a given velocity loss (VL) threshold and the maximum number of repetitions performed in that set (until failure).

**Additional file 6: Figure S3.** Individual patterns of experiencing velocity loss (VL) across the loads and testing sessions.

## Data Availability

The datasets generated and/or analysed during the current study are available in tables, Additional files and at the Open Science Framework (URL: https://osf.io/3cnw8/).

## Abbreviations

%1RM: Percentage of one-repetition maximum
CI: Confidence interval
IPIP: International Personality Item Pool
LoA: Limits of agreement
LPT: Linear position transducer
MOVER: Method of variance estimates recovery
*R*^2^: Coefficient of determination
RSE: Residual standard error
RT: Resistance training
RTF: Repetitions to failure
TOST: Two one-sided tests
VL: Velocity loss

## Footnotes

1 a We operationally defined “performing multiple repetitions within a single VL threshold” as the performance of more than a single repetition whereby all repetitions performed would “fall” under the same VL threshold. For instance, if the velocity of a given repetition in a set was exactly 20% slower than the fastest one in the set, that repetition would fall into the 20% VL threshold group. If the subsequent repetition was slower than 20%, but not slower than 25% compared to the fastest repetition in a set, that repetition would also fall into the 20% VL threshold group. In essence, performing multiple repetitions within a single VL threshold represents the ability of the participants to keep performing repetitions without experiencing considerable VL as the set progresses.
