# Supplementary material for: One Velocity Loss Threshold Does Not Fit All: Consideration of Sex, Training Status, History, and Personality Traits When Monitoring and Controlling Fatigue During Resistance Training
Source: Sports Med Open. 2023 Sep 5;9:80. doi: 10.1186/s40798-023-00626-z (PMC10480128; doi:10.1186/s40798-023-00626-z)
Supplement: Supplementary file 1 — Additional file 1: Details on the questions related to training experience and practices. [file 40798_2023_626_MOESM1_ESM.docx]

Jukic et al. (2022). One velocity loss threshold does not fit all: consideration of sex, training status, history, and personality traits when monitoring and controlling fatigue during resistance training. *Sports Medicine - Open*. Email corresponding author: ivan.jukic@aut.ac.nz. Sport Performance Research Institute New Zealand (SPRINZ), Auckland University of Technology, Auckland, New Zealand

**Supplementary file I: Details on the questions related to training experience and practices**

**How long have you been doing resistance training?**

a) 6 – 12 months

b) 1 – 2 years

c) 2 – 3 years

d) More than 3 years

**How many repetitions do you perform within your training sets, on average?**

a) 1 – 5 repetitions

b) 5 – 8 repetitions

c) 8 – 12 repetitions

d) 12 – 15 repetitions

e) More than 15 repetitions

**At what intensity of load (i.e., percentage of one repetition maximum (1RM) or your perceived maximum) do you train, on average?**

a) Lower than 60% of 1RM or perceived maximum

b) 60 – 70 % of 1RM or perceived maximum

c) 70 – 80 % of 1RM or perceived maximum

d) 80 – 90 % of 1RM or perceived maximum

e) Higher than 90% of 1RM or perceived maximum

**How many repetitions do you typically have left in reserve after you complete your training sets? (For instance, if you can perform 12 repetitions with a given load in a given exercise, but you only perform 10, you would have 2 repetitions left in reserve).**

a) 0 – 1 repetition left in reserve

b) 1 – 2 repetitions left in reserve

c) 2 – 3 repetitions left in reserve

d) 3 – 4 repetitions left in reserve

d) More than 4 repetitions left in reserve

e) I do not know or cannot estimate
