# Supplementary figures and images for: One Velocity Loss Threshold Does Not Fit All: Consideration of Sex, Training Status, History, and Personality Traits When Monitoring and Controlling Fatigue During Resistance Training
Source: Sports Med Open. 2023 Sep 5;9:80. doi: 10.1186/s40798-023-00626-z (PMC10480128; doi:10.1186/s40798-023-00626-z)

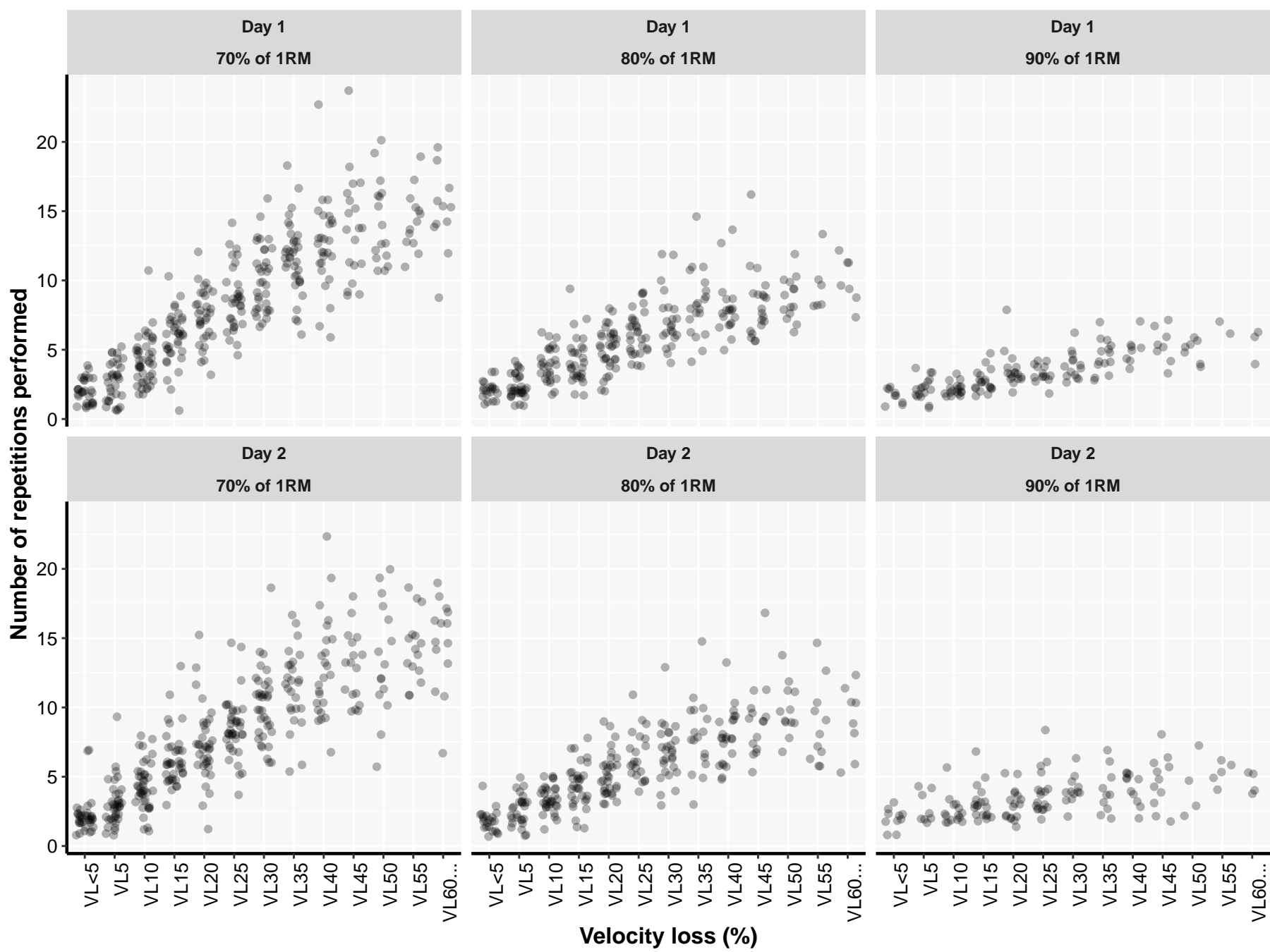

Supplement: Supplementary file 2 — Additional file 2: Figure S1. Individual repetitions and repetitions’ velocity across velocity loss (VL) thresholds. [file 40798_2023_626_MOESM2_ESM.pdf]

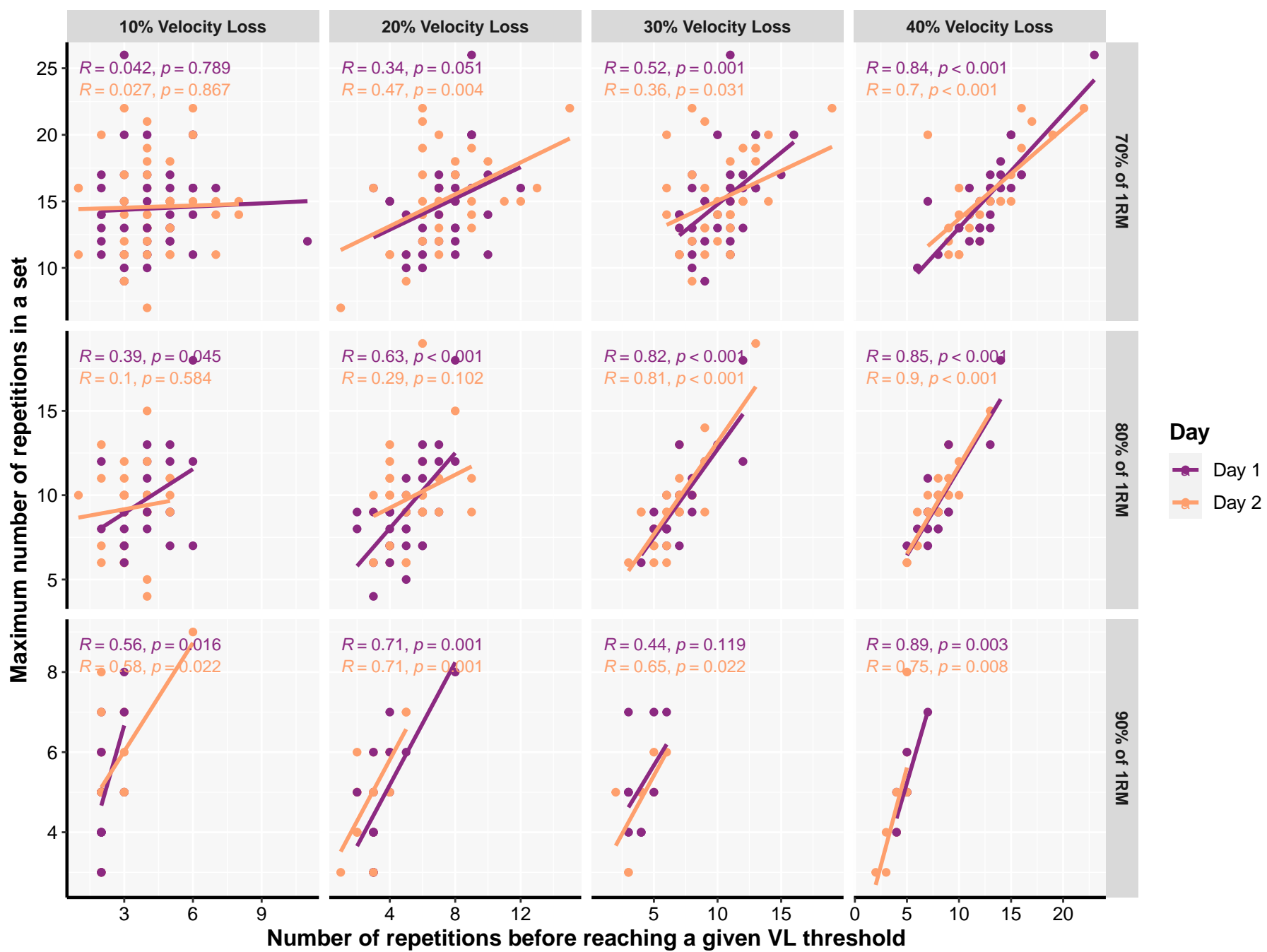

Supplement: Supplementary file 5 — Additional file 5: Figure S2. Relationships between the number of repetitions performed until reaching a given velocity loss (VL) threshold and the maximum number of repetitions performed in that set (until failure). [file 40798_2023_626_MOESM5_ESM.pdf]

Number of repetitions performed

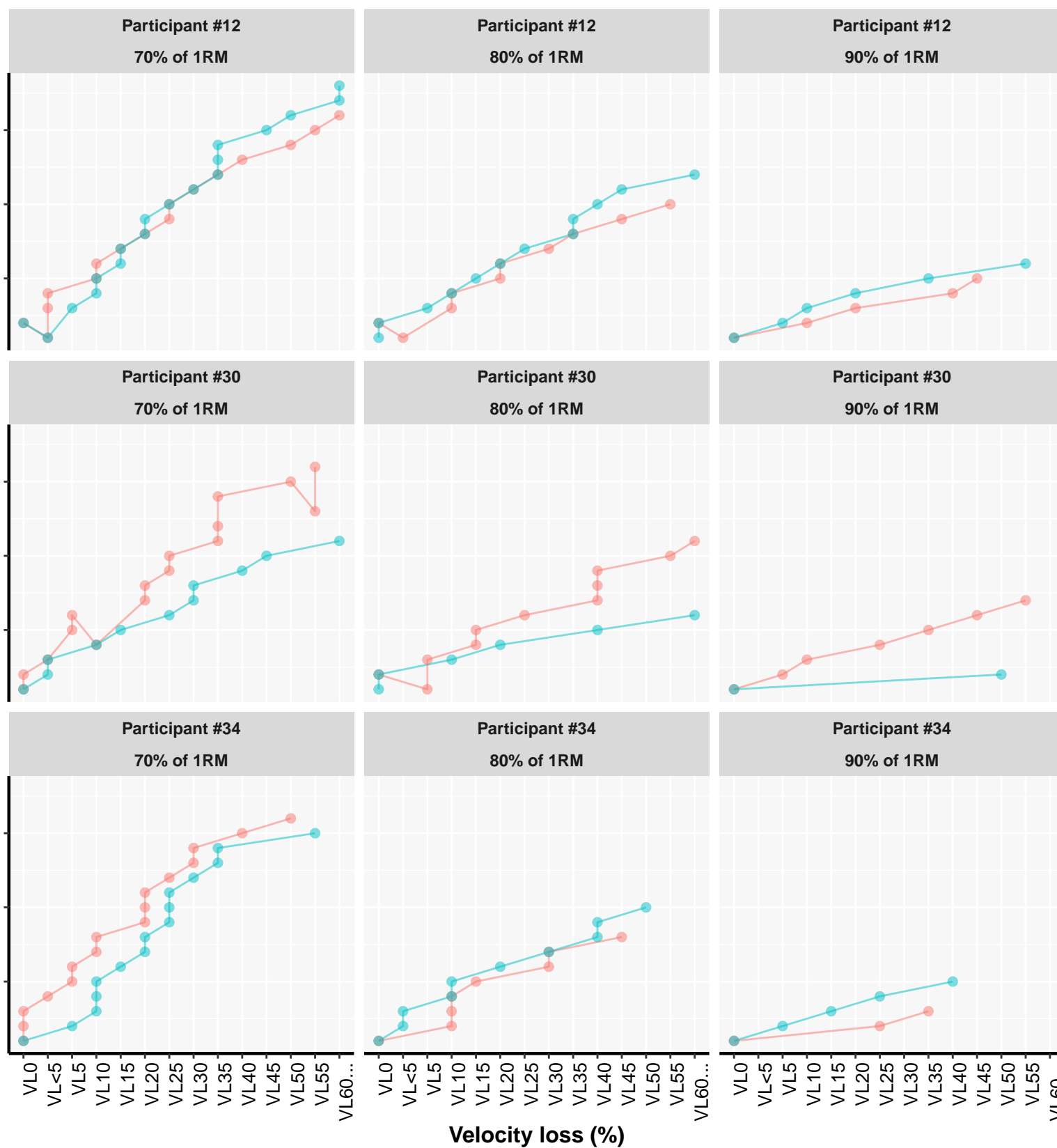

Day

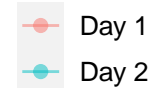

Supplement: Supplementary file 6 — Additional file 6: Figure S3. Individual patterns of experiencing velocity loss (VL) across the loads and testing sessions. [file 40798_2023_626_MOESM6_ESM.pdf]
