# Supplementary material for: One Velocity Loss Threshold Does Not Fit All: Consideration of Sex, Training Status, History, and Personality Traits When Monitoring and Controlling Fatigue During Resistance Training
Source: Sports Med Open. 2023 Sep 5;9:80. doi: 10.1186/s40798-023-00626-z (PMC10480128; doi:10.1186/s40798-023-00626-z)
Supplement: Supplementary file 3 — Additional file 3: Details on model specification and diagnostics. [file 40798_2023_626_MOESM3_ESM.docx]

Jukic et al. (2022). One velocity loss threshold does not fit all: consideration of sex, training status, history, and personality traits when monitoring and controlling fatigue during resistance training. *Sports Medicine - Open*. Email corresponding author: ivan.jukic@aut.ac.nz. Sport Performance Research Institute New Zealand (SPRINZ), Auckland University of Technology, Auckland, New Zealand

**Supplementary file III: Details on model specification and diagnostics**

Before any statistical analyses were performed, first instances when a given velocity loss threshold was reached (i.e., 5% to 60% and more in 5% increments) were identified by expressing each repetition’s velocity (in each day and with a given load) as a percentage loss from the fastest repetitions achieved in that day with a given load. For the number of repetitions outcome (both the agreement and the influential factors), the analyses were limited to the instances when the participants managed to reach the same velocity loss threshold in both days (i.e., day 1 and day 2). For instance, number of repetitions could be compared (their differences or agreement) between the days if the person performed a certain number of repetitions until reaching 20% velocity loss with a given load, but not if the participant reached 20% velocity loss on day one, but “skipped” this threshold on day 2 (e.g., reaching 15 and 25% velocity loss with a given load but not 20%). The same procedure was followed for the analysis of the mean velocity of the repetitions associated with the first instance when a given velocity loss threshold was exceeded.

Since the selection of both random and fixed effects was always theoretically motivated, the fixed effects of training practices related to participants’ repetitions left in reserve (RIR) and the loads they use during their own training were excluded from the final model since there was no reason to believe they could have affected the number of repetitions performed until reaching a given velocity loss threshold. This was especially the case given the already included fixed effect of loading condition (i.e., 70, 80, and 90% of 1RM). The Akaike Information Criterion (AIC) supported this decision as the model without these two fixed effects led to a model with greater parsimony. On the other hand, the model investigating the influential factors on the mean velocity of the repetitions associated with the first instance a given velocity loss threshold has been exceeded contained all the fixed effects since we had no reason to believe, based on some evidence, that one of the pre-defined fixed effects could be less relevant for this outcome. In this regard, the full model contained all the fixed effects, and two random effects (i.e., participants and velocity loss threshold), given that including the interaction between the two led to overfitting and convergence issues which was later quantified and confirmed by AIC. Similar situation regarding the interaction of random effects was observed – and thus procedures followed – with the general linear mixed-effects model examining the influential factors on the probability of doing multiple repetitions within a single velocity loss threshold. In addition, while we aimed to investigate what influences whether participants performed multiple repetitions within a single velocity loss threshold on day 1, but not on day 2 and vice versa, this was not done as only in 34 out of 319 cases participants were able to do multiple repetitions within a single velocity loss threshold and with the same load across two consecutive days.

For the model investigating factors affecting the probability of experiencing a 50% velocity loss in a set, the final model could not include the random effect of velocity loss threshold since, by the definition, 50% velocity loss threshold can only be reached when the individual experiences velocity loss of 50% or more. In addition, while we aimed to retain all the pre-defined fixed effects in the final model to explain this phenomenon, this was not done due to the low number of observations. We followed general and most conservative guidelines regarding the number of parameters included in the model which called for n / k = 15 (where n represents the number of observations and k represents the number of parameters). To reduce the model complexity, and thus the number of parameters in the final model, we fitted the model with all the fixed effects, and then opted to remove the predictors whose estimates had the widest confidence intervals (i.e., which were less trustworthy) until we satisfied n / k = 15, or more. This was then confirmed with the likelihood ratio test while paying particular attention to AIC scores for the full and reduced models (which were in favour for the reduced model).

Since regression-based models can be sensitive to variables that are correlated, the variance inflation factors for all predictor parameters used in the linear mixed-effects model were inspected to check for multi-collinearity. For linear mixed-effects models, a Gaussian distribution was assumed, and the approximate normal distribution of model residuals was checked to confirm goodness of fit. To ensure the assumptions of the model were met, the plotted residuals were also checked to ensure homoscedasticity prior to utilising the results of the model. To validate the assumptions of the generalised mixed-effects model, tests for uniformity of residuals, under and over dispersion, and zero-inflation were performed which confirmed the absence of significant problems with the model fit. All statistical analyses were conducted using the R language and environment for statistical computing using the *SimplyAgree^1^*, *lme4^2^*, *emmeans^3^* and *ggeffects^4^* packages, models’ performance using the *performance^5^* package, and preparation and visualisation of data using the *tidyverse^6^* and *sjPlot^7^* packages in the same computing environment.

**REFERENCES**

1. Caldwell, A.R. SimplyAgree: An R package and jamovi module for simplifying agreement and reliability analyses. *J Open Source Softw* **7**, 4148 (2022).

2. Bates, D., Mächler, M., Bolker, B. & Walker, S. Fitting linear mixed-effects models using lme4. *J Stat Softw* **67**, 1 - 48 (2015).

3. Lenth, R., Singmann, H., Love, J., Buerkner, P. & Herve, M. Emmeans: Estimated marginal means, aka least-squares means. *R package version* **1**, 3 (2022).

4. Lüdecke, D. ggeffects: Tidy data frames of marginal effects from regression models. *J Open Source Softw* **3**, 772 (2018).

5. Lüdecke, D., Ben-Shachar, M.S., Patil, I., Waggoner, P. & Makowski, D. performance: An R package for assessment, comparison and testing of statistical models. *J Open Source Softw* **6** (2021).

6. Wickham, H.*, et al.* Welcome to the Tidyverse. *J Open Source Softw* **4**, 1686 (2019).

7. Lüdecke, D. sjPlot: Data visualization for statistics in social science. *R package version* **2** (2022).
