# Supplementary material for: One Velocity Loss Threshold Does Not Fit All: Consideration of Sex, Training Status, History, and Personality Traits When Monitoring and Controlling Fatigue During Resistance Training
Source: Sports Med Open. 2023 Sep 5;9:80. doi: 10.1186/s40798-023-00626-z (PMC10480128; doi:10.1186/s40798-023-00626-z)
Supplement: Supplementary file 4 — Additional file 4: Pairwise comparisons for categorical variables with more than 2 levels for all outcomes of interest. [file 40798_2023_626_MOESM4_ESM.docx]

Jukic et al. (2022). One velocity loss threshold does not fit all: consideration of sex, training status, history, and personality traits when monitoring and controlling fatigue during resistance training. *Sports Medicine - Open*. Email corresponding author: ivan.jukic@aut.ac.nz. Sport Performance Research Institute New Zealand (SPRINZ), Auckland University of Technology, Auckland, New Zealand

**Supplementary file III: Pairwise comparisons for significant, categorical variables with more than 2 levels for all outcomes of interest.**

Table 1. Pairwise comparisons with Holm-Bonferroni correction for the effects of loads on the number of repetitions completed across velocity loss thresholds

| *contrast* | *estimate* | *SE* | *t.value* | *p.value* |
| --- | --- | --- | --- | --- |
| 70% / 80% | 2.13 | 0.13 | 15.83 | < 0.001 |
| 70% / 90% | 4.00 | 0.23 | 17.78 | < 0.001 |
| 80% / 90% | 1.87 | 0.24 | 7.92 | < 0.001 |

Table 2. Pairwise comparisons with Holm-Bonferroni correction for the effects of loads on the velocity of repetitions associated with the first instance when a given velocity loss threshold was exceeded

| *contrast* | *estimate* | *SE* | *t.value* | *p.value* |
| --- | --- | --- | --- | --- |
| 70% / 80% | 0.07 | 0.00 | 35.84 | < 0.001 |
| 70% / 90% | 0.14 | 0.00 | 44.78 | < 0.001 |
| 80% / 90% | 0.07 | 0.00 | 22.05 | < 0.001 |

Table 3. Pairwise comparisons with Holm-Bonferroni correction for the effects of loads on the probability of not reaching the same velocity loss threshold in two consecutive days

| *contrast* | *odds.ratio* | *SE* | *z.value* | *p.value* |
| --- | --- | --- | --- | --- |
| 70% / 80% | 0.41 | 0.06 | -6.00 | < 0.001 |
| 70% / 90% | 0.12 | 0.03 | -10.29 | < 0.001 |
| 80% / 90% | 0.30 | 0.06 | -5.93 | < 0.001 |

Table 4. Pairwise comparisons with Holm-Bonferroni correction for the effects of (participants’) training practices related to the number of repetitions one typically performs during training on the probability of performing multiple repetitions within a single velocity loss threshold

| *contrast* | *odds.ratio* | *SE* | *z.value* | *p.value* |
| --- | --- | --- | --- | --- |
| 1 – 8 / 8 – 12 | 1.06 | 0.18 | 0.34 | 0.740 |
| 1 – 8 / more than 12 | 0.56 | 0.10 | -3.17 | < 0.001 |
| 8 – 12 / more than 12 | 0.53 | 0.09 | -3.80 | < 0.001 |

Table 5. Pairwise comparison with Holm-Bonferroni correction for the effects of loads on the probability of performing multiple repetitions within a single velocity loss threshold

| *contrast* | *odds.ratio* | *SE* | *z.value* | *p.value* |
| --- | --- | --- | --- | --- |
| 70% / 80% | 2.37 | 0.30 | 6.85 | < 0.001 |
| 70% / 90% | 9.60 | 2.10 | 10.32 | < 0.001 |
| 80% / 90% | 4.05 | 0.91 | 6.20 | < 0.001 |

Table 6. Pairwise comparisons with Holm-Bonferroni correction for the effects of loads on the probability of experiencing 50% velocity loss in a set

| *contrast* | *odds.ratio* | *SE* | *z.value* | *p.value* |
| --- | --- | --- | --- | --- |
| 70% / 80% | 1.83 | 1.18 | 0.93 | 0.350 |
| 70% / 90% | 31.35 | 28.29 | 3.82 | < 0.001 |
| 80% / 90% | 17.16 | 13.92 | 3.51 | < 0.001 |
